# Neutrophil-mediated immune dysregulation in recurrent miscarriage: implications of APP–CD74 signaling and CXCL1 as diagnostic biomarkers

**DOI:** 10.3389/fimmu.2025.1707497

**Published:** 2025-12-04

**Authors:** Shan He, Fei Ma, Meng Yu, Jia-man Wu, You-ming Gong, Ke-wang Luo, Hong Chang, Qi Liang, Yan Ning

**Affiliations:** 1 Shenzhen Maternity and Child Healthcare Hospital, Women and Children’s Medical Center, Southern Medical University, Shenzhen, Guangdong, China; 2 Guangzhou University of Chinese Medicine, Guangzhou, Guangdong, China; 3 Guangdong Provincial Hospital of Chinese Medicine, The Second Affiliated Hospital of Guangzhou University of Chinese Medicine, Guangzhou, Guangdong, China; 4 Shenzhen Bao’an Traditional Chinese Medicine Hospital, Guangzhou University of Chinese Medicine, Shenzhen, Guangdong, China

**Keywords:** recurrent miscarriage (RM), neutrophil activation, APP–CD74, CXCL1, immune dysregulation, TNF-α-polarized, single-cell, biomarker

## Abstract

**Background:**

Recurrent miscarriage (RM), characterized by the loss of two or more consecutive pregnancies, is a significant challenge in reproductive medicine. While immunological dysregulation is increasingly recognized as a key contributor, the role of innate immunity, particularly neutrophils, remains underexplored. Understanding neutrophil-driven immune mechanisms may provide novel diagnostic and therapeutic insights for RM.

**Methods:**

We integrated bulk transcriptomic datasets from RM, autoimmune diseases, and decidua single-cell RNA sequencing data. Differentially expressed genes (DEGs) and enriched pathways were identified, and intercellular communication was analyzed using CellChat. Key genes (KGs) were validated through ROC analysis, nomogram modeling, and experimental validation in trophoblast cells.

**Results:**

Single-cell profiling revealed increased neutrophil proportions and distinct activation states in RM. Neutrophils exhibited a TNF-α-driven polarized phenotype with upregulated oxidative stress and antigen presentation. Intercellular communication analysis identified the APP-CD74 axis as a key signaling pathway. CXCL1 and related genes were consistently upregulated and showed strong diagnostic performance. Molecular docking identified potential therapeutic compounds targeting CXCL1, and *in vitro* assays confirmed immune-mediated trophoblast injury.

**Conclusions:**

Neutrophil-driven immune dysregulation, with TNF-α-polarized neutrophils and APP–CD74 signaling, contributes to RM pathogenesis. CXCL1 and related genes serve as promising biomarkers, offering new opportunities for early diagnosis and therapeutic intervention.

## Introduction

Recurrent miscarriage (RM), defined as the loss of two or more consecutive pregnancies before 24 weeks, affects around 3% of women of reproductive age, leading to significant psychological and public health challenges (1). While maternal anatomical abnormalities, endocrine disorders, and chromosomal anomalies contribute to RM, more than half of cases remain unexplained, highlighting the need to better understand the underlying mechanisms (2, 3).

Evidence suggests that immune dysregulation at the maternal–fetal interface contributes significantly to RM. While much research has focused on adaptive immune mechanisms, such as regulatory T cells and uterine natural killer (NK) cells, innate immune pathways—especially those involving neutrophils—have been less explored (4, 5).

Neutrophils are traditionally characterized as short-lived antimicrobial responders; yet, accumulating evidence highlights their functional diversity, including roles in cytokine release, antigen presentation, tissue remodeling, and crosstalk with both immune and non-immune cells (6). Despite these insights, the contribution, heterogeneity, and activation states of neutrophils in RM remain poorly defined, representing an important knowledge gap. In addition, the lack of mechanistically informed biomarkers has hindered early detection and personalized management of RM.

To address these limitations, we integrated single-cell RNA sequencing with bulk transcriptomics to examine neutrophil dynamics and inflammatory signaling in RM. We further examined ligand–receptor communication, exploring APP–CD74 as a potential signaling axis, and identified neutrophil-associated genes such as CXCL1 as candidate diagnostic biomarkers. Complementary *in-vitro* assays using an inflammatory trophoblast model were employed to provide indirect support for immune activation–associated trophoblast stress; while these experiments do not directly assess neutrophil–trophoblast interactions, they recapitulate key inflammatory features relevant to RM pathophysiology. Taken together, this study aims to advance understanding of neutrophil-mediated immune dysregulation in RM and to inform the development of potential diagnostic and therapeutic strategies.

## Materials and methods

### Bulk transcriptomic data acquisition and preprocessing

All transcriptomic datasets were obtained from the Gene Expression Omnibus (GEO; https://www.ncbi.nlm.nih.gov/geo/). The RM discovery cohort comprised GSE113790 (3 RM vs. 3 control endometrium; Illumina HiSeq 2000, GPL11154), GSE183555 (5 RM vs. 5 control endometrium; NextSeq 550, GPL21697), and GSE26787 (5 RM vs. 5 control endometrium; Affymetrix HG-U133_Plus_2, GPL570). The independent validation cohort included GSE165004 (24 RM vs. 24 control endometrium).

Autoimmune disease (AID) datasets used for cross-disease comparisons included rheumatoid arthritis (RA: GSE225731, GSE89408), systemic lupus erythematosus (SLE: GSE50772, GSE81622), and systemic sclerosis (SSc: GSE95065, GSE9285). Raw data (CEL files) or processed matrices were imported according to platform requirements, with probe IDs mapped to official gene symbols using the corresponding GPL annotations.

### Batch effect correction (within-cohort)

To minimize technical variation while preserving disease-specific biology, normalization and batch correction were conducted within each disease cohort rather than across all datasets simultaneously. For cohorts integrating multiple platforms/studies (e.g., RM discovery set; RA; SLE; SSc), we applied ComBat from the sva package (v3.50.0) followed by limma (7) (v3.50.0) empirical Bayes modeling. Correction efficacy was verified by principal component analysis (PCA) to ensure removal of batch-driven structure without over-correction.

### Stratified integration and cross-disease comparative analysis

**Figure 1:**
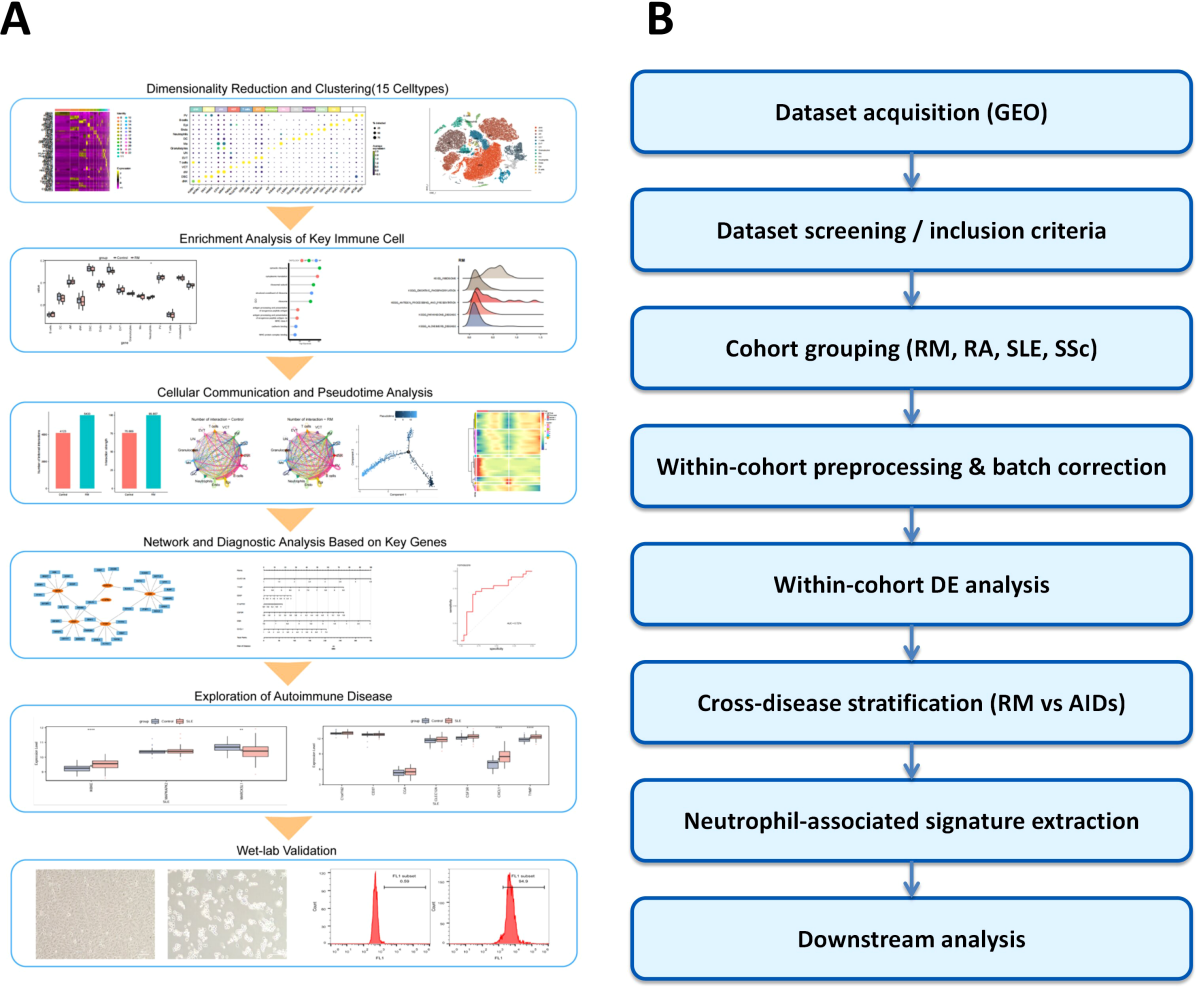
Study workflow. **(A)** Overview of the workflow integrating bulk transcriptomic and single-cell RNA-seq data to analyze immune dysregulation in recurrent miscarriage (RM). **(B)** Bulk transcriptomic preprocessing and stratified analysis. GEO datasets were screened by disease type (RM, RA, SLE, SSc), and differential expression (DE) analysis was performed within each cohort. Cross-disease stratification identified RM-specific neutrophil-related signatures for downstream analysis.

To distinguish RM-specific neutrophil signatures from general autoimmune-associated inflammation, analyses followed a two-stage, stratified workflow:

- Within-cohort analyses: Differential expression (DE) and pathway enrichment were first performed separately in each disease cohort under identical thresholds (|log_2_FC| > 0.5, *p* < 0.05) to derive cohort-level neutrophil-related signatures.
- Cross-disease stratification: RM-associated DEGs were then compared against AID results (RA, SLE, SSc). Transcripts consistently dysregulated across AIDs were flagged as “shared autoimmune” and excluded from RM-specific interpretation, thereby isolating RM-preferential neutrophil activation patterns. Candidate markers were further checked against single-cell–derived neutrophil DEGs from decidual tissue to maintain cell-type specificity and biological plausibility. A schematic overview of dataset inclusion, within-cohort preprocessing, and cross-disease stratification has been added to **Figure 1**.

### Single-cell transcriptomic data processing

Publicly available scRNA-seq data from RM decidua (GEO: GSE214607, comprising 5 normal and 3 RM samples) (8) were processed using Seurat (9) (v4.2.0). Quality control excluded cells expressing <200 genes, removed undetected genes, and retained cells with <7,000 expressed genes, <15% mitochondrial gene content, <5% hemoglobin gene content, and <40,000 UMIs. Data underwent log-normalization (NormalizeData). Highly variable genes were identified through mean-variance relationship modeling, enabling PCA on scaled data. Graph-based clustering at resolution 0.4 (FindClusters; shared nearest neighbor optimization) applied to 13 significant PCs yielded 23 clusters. Dimensionality reduction was performed via t-SNE (RunTSNE), with cluster identities determined using canonical lineage markers followed by quantitative assessment of cell type proportions. All analyses adhered to original public deposition ethics protocols.

### Cell communication analysis and ligand-receptor expression

CellChat objects were created separately for the RM and Control groups using the CellChat R package (version 1.1.3, https://www.github.com/sqjin/CellChat) (10), based on UMI count matrices per group. Intercellular communication analysis utilized the “CellChatDB.human” ligand-receptor interaction database as reference, applying default parameters. The “mergeCellChat” function integrated group-specific CellChat objects, enabling comparative assessment of total interaction counts and strength. Differential interactions between cell types across groups were visualized using the “netVisual_diffInteraction” function. Signaling gene expression distributions were displayed between groups via the “netVisual_bubble” and “netVisual_aggregate” functions.

### Pseudotime analysis for cellular trajectories

Cellular trajectories were constructed through pseudotime analysis using Monocle 2 (11). This algorithm employs reversed graph embedding on specified gene lists to generate pseudotime plots that reveal branching events and differentiation pathways. Raw count data were normalized by estimating size factors for trajectory reconstruction. Highly variable and expressed genes, with dispersion estimates ≥1 and mean expression ≥0.1, established pseudotime trajectories to capture cellular heterogeneity and differentiation dynamics (12). DDRTree algorithm parameters within Monocle 2 remained at default settings 2. Branch expression analysis modeling (BEAM) integrated into Monocle 2 identified significantly branch-dependent genes (11). Branch-dependent expression patterns were visualized as heatmaps using the same platform.

### Single-cell transcription factor analysis

Cellular heterogeneity within tissues originates from differences in transcriptional states, established and maintained by complex Gene Regulatory Networks (GRNs) orchestrated by TFs. Analysis of GRNs at single-cell resolution enables examination of biological mechanisms driving this heterogeneity, providing insights relevant to disease diagnostics, therapeutic development, and developmental differentiation.

### Differentially expressed genes

To identify genes differentially expressed between RM and control samples, differential expression analysis was conducted using the R package limma (7) on an integrated and batch-corrected RM cohort derived from datasets GSE113790, GSE183555, and GSE26787. Gene selection utilized thresholds of |log_2_ fold change (log_2_FC)| > 0.5 and a *p*-value < 0.05. These identified DEGs proceeded to downstream analyses.

### Gene ontology and Kyoto encyclopedia of genes and genomes enrichment analysis

Functional enrichment analysis was conducted to understand the biological context of the target gene set. GO enrichment (13), comprising Biological Processes (BP), Molecular Functions (MF), and Cellular Components (CC), was performed along with pathway enrichment analysis using the KEGG database (14). Both analyses were executed using the R package clusterProfiler (15) (v 4.2.2).

### Gene set variation analysis

To evaluate the relative activity levels of predefined gene sets across individual samples, GSVA was performed using the R package GSVA (16, 17) (v 1.42.0). The gene expression matrix from RM samples was converted into a sample-wise gene set enrichment score matrix based on the Hallmark gene sets from the Molecular Signatures Database (MSigDB; https://www.gsea-msigdb.org/gsea/msigdb/). These enrichment scores represent the relative expression level of each gene set within each sample. Subsequently, differential activity analysis of these gene sets between the RM and control cohorts was conducted using empirical Bayes moderated t-tests from the limma package, identifying sets showing significant changes associated with disease progression.

### Gene set enrichment analysis

GSEA was utilized to determine whether predefined gene sets demonstrate statistically significant, concordant differences in expression between the RM and control phenotypes (18). This method examines the distribution pattern of a gene set’s members within a rank-ordered list of all genes based on their differential expression magnitude (|log_2_FC|). Using the ranked gene list derived from the differential expression analysis between sample groups, GSEA was performed with the R package clusterProfiler. The analysis employed the following parameters: 1000 permutations for significance estimation, a minimum gene set size of 10, a maximum size of 500, and Benjamini-Hochberg (BH) adjustment for multiple testing. Enrichment analysis was conducted specifically against the KEGG pathway gene sets curated within the MSigDB database (16–18), with a significance threshold set at a *p*-value < 0.05.

### Deconvolution-based infiltration analysis

Single-sample Gene Set Enrichment Analysis (ssGSEA) (19), an extension of the standard GSEA methodology, was applied to calculate sample-specific enrichment scores for individual gene sets. Each ssGSEA score indicates the degree of coordinated up- or down-regulation shown by the genes within a specific set in a given sample. Based on prior single-cell annotation results, relative enrichment scores were calculated for each predefined cell type. This process involved first determining the relative enrichment score per cell based on the expression profiles of the top 50 most significantly DEGs identified for that specific cell type. These per-cell scores were then aggregated to measure the relative enrichment level of each cell type within the gene expression profile of each RM sample. Differential cellular infiltration levels across sample groups were visualized using the R package ggplot2 (20) (v 3.3.6).

### Immune response enrichment analysis

The IREA approach, developed by Ang Cui et al. (21), was implemented to determine cytokine activities and immune cell polarization states from gene expression data across various immunological contexts. This cell-centric methodology characterizes over 66 cytokine-driven polarization states within immune cell types, including previously uncharacterized states. Using this framework, IREA was applied to identify the dominant cytokines eliciting responses from key immune cell types, while assessing their polarization states within the RM condition. This analysis utilized DEGs (|log_2_FC| > 0.5, *p* < 0.05) identified within these key immune cell types between RM and normal control groups within our scRNA-seq dataset.

### GeneMANIA network analysis

The GeneMANIA resource (http://genemania.org) utilizes an integrative algorithm to predict functional associations between target genes of interest and biologically related partners (22). This includes identification of protein-protein interactions, protein-DNA binding events, shared pathway membership, co-expression patterns, physical co-localization, and correlated physiological/biochemical responses. Using this platform, a comprehensive protein-protein interaction (PPI) network was constructed around the identified key genes (KGs) central to our study.

### Receiver operating characteristic analysis

ROC curves were generated using the R package pROC (23) (v 1.18.5) to assess the diagnostic potential of KGs for RM. This method, formally known as ROC analysis, provides an integrated performance metric depicting the dynamic relationship between sensitivity and specificity across all classification thresholds. The primary quantitative measure, the Area Under the Curve (AUC), was calculated from these plots to evaluate biomarker efficacy. AUC values range between 0.5 (no discriminative capacity) and 1.0 (perfect diagnostic accuracy), with higher values indicating superior predictive performance.

### Diagnostic nomogram construction and validation

A clinical prediction model was developed using the R package rms (v 6.5-0) to construct a diagnostic nomogram for RM risk stratification. This visual prognostic tool calculates predictive scores based on expression levels of KGs, with the composite risk score derived from the summation of all individual gene-specific point assignments. The discriminative capacity of the resultant risk score for RM detection was subsequently evaluated through ROC curve analysis.

### RNA-binding protein-mRNA network construction

This investigation utilized the widely-used open-source platform StarBase (https://starbase.sysu.edu.cn/tutorialAPI.php#RBPTarget) for comprehensive RNA interactome profiling. This resource integrates CLIP-seq, degradome-seq, and RNA-RNA interaction data to characterize associations between mRNA expression and RBP activity. Significant mRNA-RBP interactions were identified using a dual-filter threshold (*p* < 0.05 and clusterNum ≥ 5), defining interactions containing ≥5 binding sites as high-confidence pairs. The regulatory network was visualized using Cytoscape (v 3.9.1).

### mRNA-TF network construction

As key regulators recognizing specific DNA sequences, TFs coordinate transcriptional programs governing diverse biological processes. This study examined the TRRUST database (https://www.grnpedia.org/trrust/), a manually curated resource documenting human transcriptional regulatory networks comprising 800 TFs and 8,444 target relationships with directional annotations (activation/repression). The analysis systematically extracted TF-interactor pairs for KGs and constructed a comprehensive transcriptional regulatory network, which was subsequently visualized using Cytoscape.

### Molecular docking

Drug-gene interactions for KGs were initially identified via the DGIdb platform (https://www.dgidb.org/), with candidate compound structures retrieved from PubChem in SDF format and converted to mol2 using OpenBabel (v 2.4.1). Ligand preparation involved processing these mol2 files in AutoDockTools (v 1.5.7) through torsion assignment and pdbqt conversion. Concurrently, experimentally resolved protein structures underwent solvent stripping and cofactor removal in PyMOL (v 2.5.0), followed by polar hydrogen addition and pdbqt conversion in AutoDockTools. Docking simulations employed the Lamarckian genetic algorithm, where binding energy (ΔG, kcal/mol) served as the primary affinity metric—lower values indicating enhanced steric complementarity, greater thermodynamic stability, and higher binding probability. Final binding poses were validated and visualized in PyMOL.

### Cell culture and reactive oxygen species measurement by flow cytometry

HTR-8/Svneo cells were provided by the Chinese Academy of Sciences Cell Bank (China). They were cultured in RPMI-1640 medium (Wuhan Servicebio, G4531-500mL) supplemented with 10% fetal bovine serum (FBS) (Shanghai Chunqiu, HB-FBS-50) and 1% penicillin-streptomycin at 37 °C in a humidified atmosphere with 5% CO_2_. To establish an immune-mediated RM model, cells were treated with anti-β_2_-glycoprotein I (anti-β_2_-GPI) antibody (US Biological, 031990-Ml650) at a 1:100 dilution for 48 hours, to mimic the inflammatory environment associated with antiphospholipid syndrome, a known contributor to RM, while untreated cells served as controls.

Intracellular ROS levels were measured using the DCFH-DA probe (Beyotime, S0033). After treatment, cells were trypsinized (Amresco, 0457), washed with PBS (Servicebio, G0002), and resuspended at a density of 1×10^6^–2×10^6^ cells/mL. Cells were incubated with 10 µM DCFH-DA for 20 min at 37 °C in the dark, with gentle mixing every 3–5 min. Following incubation, cells were washed three times with cold PBS and analyzed using a Beckman flow cytometer (A00-1-1102) with excitation at 488 nm and emission at 525 ± 20 nm. Data were collected and analyzed with FlowJo software. ROS-positive cells were identified in the FL1 channel, and the results were expressed as the percentage of cells in the FL1-positive subset (% ROS-positive cells) rather than mean fluorescence intensity.

Cell morphology was assessed in parallel using a fluorescence inverted microscope (Shanghai Nikon, TE300). Representative images of the control and model groups were captured, with particular attention to cellular aggregation and reduction in cell number, which were considered indicative of immune-induced cell damage.

### Statistical analysis

Statistical analysis was performed using R (v 4.3.3). Spearman’s rank correlation test was used to assess correlations between variables. For comparisons between two groups, Wilcoxon signed-rank test was applied, and for comparisons across three or more groups, Kruskal-Wallis test was used. A two-tailed p-value of less than 0.05 was considered statistically significant. For wet-lab experiments, including flow cytometry and cell morphology assays, data are presented as mean ± standard deviation (SD). Statistical significance between two groups was assessed using a two-tailed Student’s t-test, while one-way ANOVA with Tukey’s *post hoc* test was used for multiple comparisons. All analyses were performed with GraphPad Prism 9 and R, with statistical significance set at *p* < 0.05.

## Results

### Single-cell dimensionality reduction, clustering, and annotation

The overall workflow of this work is illustrated in **Figure 1**.

**Figure 2:**
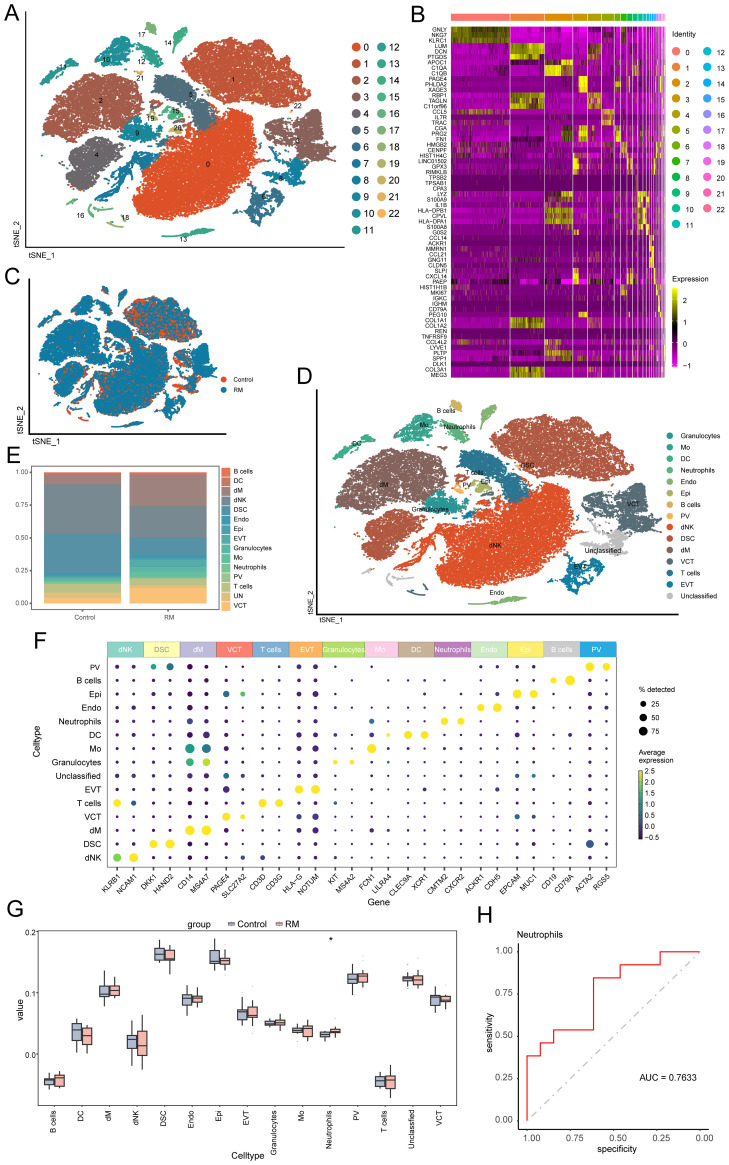
Single-cell profiling identifies 15 decidual cell populations and neutrophil enrichment in RM. **(A)** t-SNE plot showing 23 transcriptionally defined cell clusters. **(B)** Heatmap of representative DEGs for each cluster. **(C)** t-SNE plot comparing RM vs. control sample distributions. **(D)** Cell-type annotation of clusters using canonical lineage markers. **(E)** Barplot comparing the proportions of 15 major decidual cell types between RM and controls. **(F)** Marker-gene expression heatmap used to confirm cell-type identities. Neutrophil and Granulocyte clusters were defined as transcriptionally distinct subpopulations. Neutrophils exhibited high CMTM2, CXCR2 expression consistent with mature activation states, whereas Granulocytes expressed KIT, MS4A2, characteristic of immature granulocytic cells. **(G)** Boxplot showing proportional abundance differences of the 15 cell types. **(H)** ROC curve evaluating the diagnostic performance of neutrophil abundance (AUC > 0.7). Asterisks denote statistical significance: *p < 0.05.

To characterize cellular origins of highly expressed genes, the analysis examined the scRNA-seq dataset GSE214607 from RM samples (24, 25). After quality control, 57,238 cells from eight samples underwent graph-based clustering, revealing 23 distinct clusters (**Figure 2A**). Cluster-specific significantly DEGs were visualized via heatmap in **Figure 2B**) (detailed in **Supplementary Table S1**). Cellular distribution across RM and control groups was mapped using t-SNE (**Figure 2C**). Cell types were annotated via lineage-specific biomarkers (**Figure 2D**), **Supplementary Table S2**), identifying 15 populations: decidual macrophages (dM), neutrophils, decidual natural killer cells (dNK), decidual stromal cells (DSC), extravillous trophoblasts (EVT), villous cytotrophoblasts (VCT), monocytes (Mo), endothelial cells (Endo), dendritic cells (DC), perivascular cells (PV), epithelial glandular cells (Epi), Granulocytes, B cells, T cell, and unclassified cells. Comparative proportional distributions of these populations between cohorts are shown in **Figure 2E**), with canonical marker gene expression per cell type illustrated in **Figure 2F**). To clarify potential overlap between granulocytes and neutrophils, we further examined their marker expression profiles. The “Neutrophil” cluster was characterized by high expression of CMTM2 and CXCR2, consistent with mature, activated neutrophils exhibiting chemotactic and pro-inflammatory functions. In contrast, the “Granulocyte” cluster expressed KIT and MS4A2, markers indicative of an immature or alternative granulocytic lineage, possibly representing basophil- or mast-cell-like precursors. These two clusters therefore represent transcriptionally and functionally distinct populations rather than overlapping subsets. Accordingly, neutrophils were not included within the broader granulocyte cluster but defined as a separate lineage based on these canonical markers and differential gene-expression signatures (**Figures 2D, F**).

### Cell-type deconvolution

TF analysis of scRNA-seq data revealed cell type-specific TF expression patterns (**Supplementary Figure S1**). Signature TFs across samples and groups are visualized in **Supplementary Figures S1B, C**.

Using DEGs per cell type (**Supplementary Table S3**), we performed deconvolution of the 15 identified cell populations into bulk transcriptomic RM cohorts (GSE113790, GSE183555, GSE26787). Comparative analysis revealed significantly higher proportions of neutrophils in RM samples compared to controls (*p* < 0.05; **Figure 2G**). ROC curve analysis confirmed the diagnostic potential of neutrophil abundance for RM (AUC > 0.7; **Figure 2H**). The consistent enrichment and diagnostic potential of neutrophils across independent datasets underscore their clinical relevance in RM.

### Functional enrichment analysis of neutrophils

Differential gene expression analysis was conducted on neutrophils between sample groups (**Supplementary Table S4**; n=386 DEGs, *p* < 0.05, |Log_2_FC| > 0.5). A heatmap illustrates the top 20 significantly DEGs in RM patients compared to the control group (**Supplementary Figure S2A**).

To understand the biological functions of these DEGs and the mechanisms distinguishing the groups, we performed GO term (**Supplementary Table S5**), KEGG pathway (**Supplementary Table S6**), GSEA (**Supplementary Table S7**), and GSVA (**Supplementary Table S8**) enrichment analyses. Significantly enriched pathways are shown in **Supplementary Figures S2B–E**.

GO enrichment analysis identified significant enrichment for BP including cytoplasmic translation, antigen processing and presentation of exogenous peptide antigen, antigen processing and presentation of exogenous peptide antigen via MHC class II. Enriched CC included cytosolic ribosome, ribosomal subunit, ribosome. MF showing enrichment structural constituent of ribosome, cadherin binding, MHC protein complex binding (**Supplementary Figure S2B**). KEGG pathway analysis revealed enrichment in pathways including Coronavirus disease - COVID-19, Ribosome, Phagosome (**Supplementary Figure S2C**).

Using the MSigDB database, we identified the most significantly enriched signaling pathways based on their Normalized Enrichment Score (NES). GSEA showed significant enrichment in the RM group for pathways including RIBOSOME, OXIDATIVE PHOSPHORYLATION, ANTIGEN PROCESSING AND PRESENTATION, PARKINSONS DISEASE, and ALZHEIMERS DISEASE (**Supplementary Figure S2D**).

The activation status of specific pathways in RM, as evaluated by GSVA, demonstrated significant upregulation in SYSTEMIC LUPUS ERYTHEMATOSUS, ANTIGEN PROCESSING AND PRESENTATION, LYSOSOME, ASTHMA, and INTESTINAL IMMUNE NETWORK FOR IGA PRODUCTION pathways (**Supplementary Figure S2E**).

**Figure 3:**
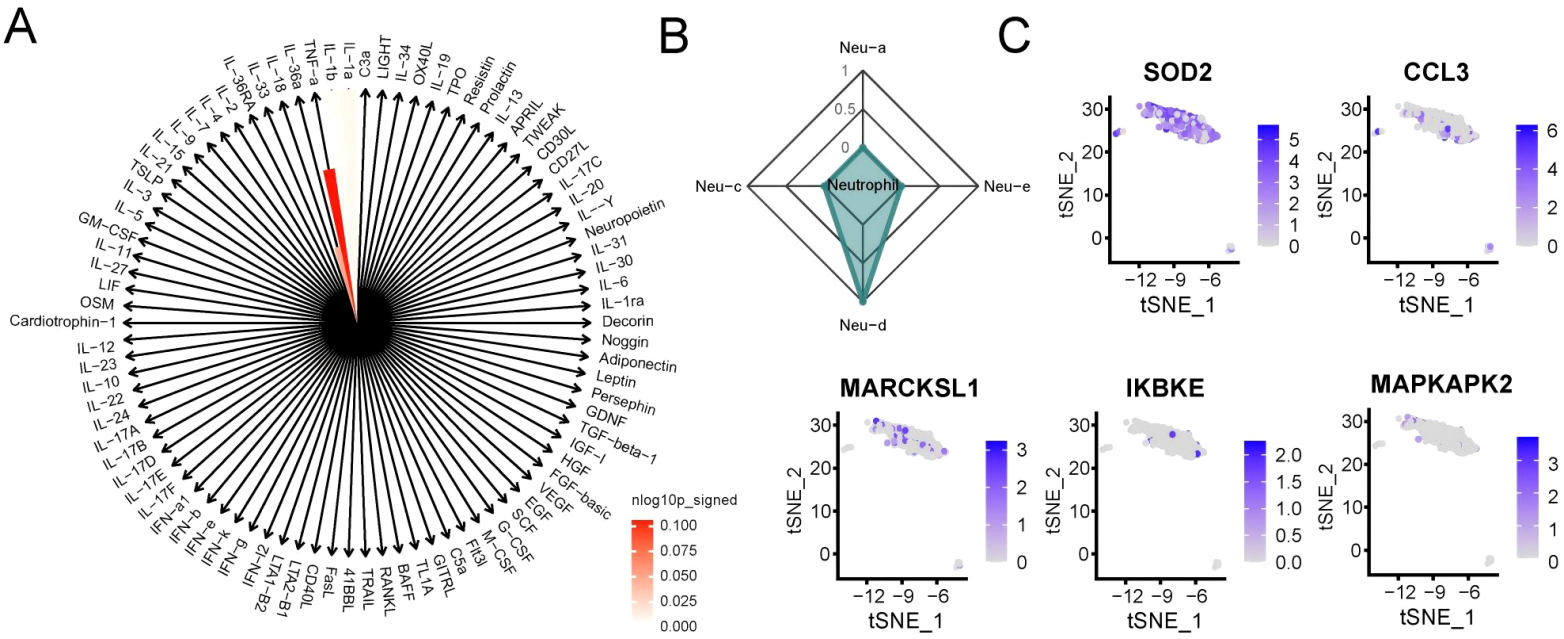
Neutrophils in RM exhibit TNF-α–driven polarized activation. **(A)** Cytokine enrichment plot showing dominant TNF-α signaling in RM neutrophils. **(B)** Radar plot showing the IREA-derived Neu-d polarized state in RM. Neutrophil polarization signatures (Neu-a to Neu-d) were defined according to the IREA cytokine-response atlas. RM samples showed pronounced Neu-d (TNF-α–driven) enrichment compared with controls. **(C)** Expression levels of Neu-d marker genes (SOD2, CCL3, MAPKAPK2, MARCKSL1, IKBKE) in neutrophils.

To characterize the immune activation state of neutrophils in RM, we conducted IREA based on significantly upregulated DEGs in RM. The cytokine enrichment plot showed that neutrophils in RM were predominantly enriched for cytokines such as TNF-α (**Figure 3A**). Additionally, cell polarization radar plot analysis confirmed that neutrophils in RM primarily exhibited a TNF-α-induced polarized state (Neu-d), a phenotype potentially amplifying inflammatory responses in RM (**Figure 3B**). Analysis of established Neu-d marker genes (SOD2, CCL3, MAPKAPK2, MARCKSL1, IKBKE) expression within neutrophils revealed significantly elevated expression of SOD2 (**Figure 3C**).

Based on the IREA framework, four neutrophil polarization signatures (Neu-a to Neu-d) were inferred according to cytokine-response reference gene sets. Among these, Neu-d represents a TNF-α–driven pro-inflammatory phenotype associated with oxidative-stress and antigen-presentation activities. In our analysis, RM samples showed strong enrichment of the Neu-d signature, whereas control decidual neutrophils predominantly exhibited Neu-a/b/c patterns, reflecting a balanced, non-inflammatory state. This result highlights a TNF-α-dominated polarization shift in RM.

Together, these results indicate that neutrophils in RM adopt a TNF-α–driven polarized phenotype, characterized by enhanced oxidative stress and antigen presentation activity.

### Intercellular communication analysis

**Figure 4:**
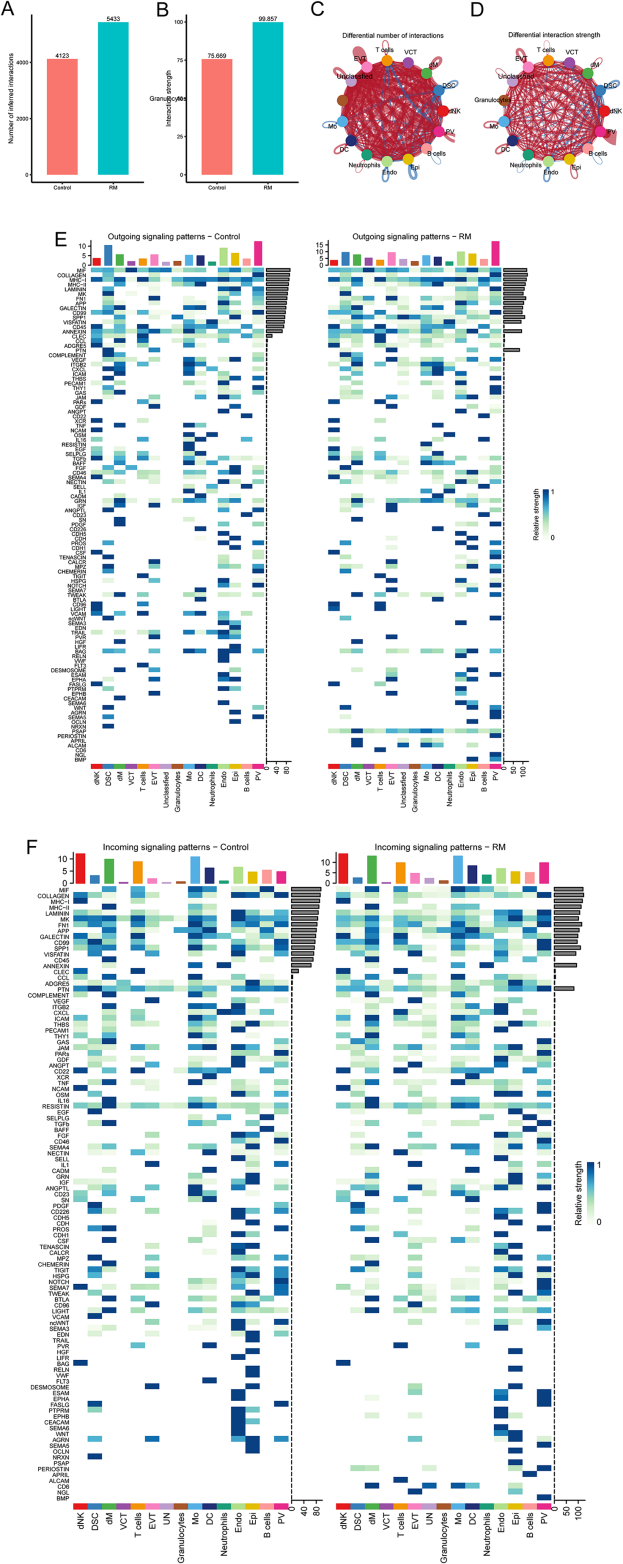
Cell–cell communication analysis highlights endothelial–neutrophil APP–CD74 signaling in RM. **(A)** Number of interactions across cell types in RM vs. controls. **(B)** Interaction strength across cell types. **(C)** Network plot showing differential interaction numbers. **(D)** Network plot showing differential interaction strengths. **(E)** Outgoing signaling patterns of each cell type. **(F)** Incoming signaling patterns of each cell type.

To examine the interaction between neutrophils and other cell types in RM, we utilized the R package ‘CellChat’ to identify changes in intercellular crosstalk. As shown in **Figures 4A, B**, the total number and strength of overall cell-cell interactions were significantly increased in the RM group compared to the NC group. The network plot demonstrates that the intensity of most interactions increased across cell types, except for interactions between T cells and DSC, and between Mo and PV (**Figures 4C, D**). Comparison of signaling patterns emitted and received by individual cell types (**Figures 4E, F**) revealed significantly enhanced strength of pathway signaling received by neutrophils in the RM group.

**Figure 5:**
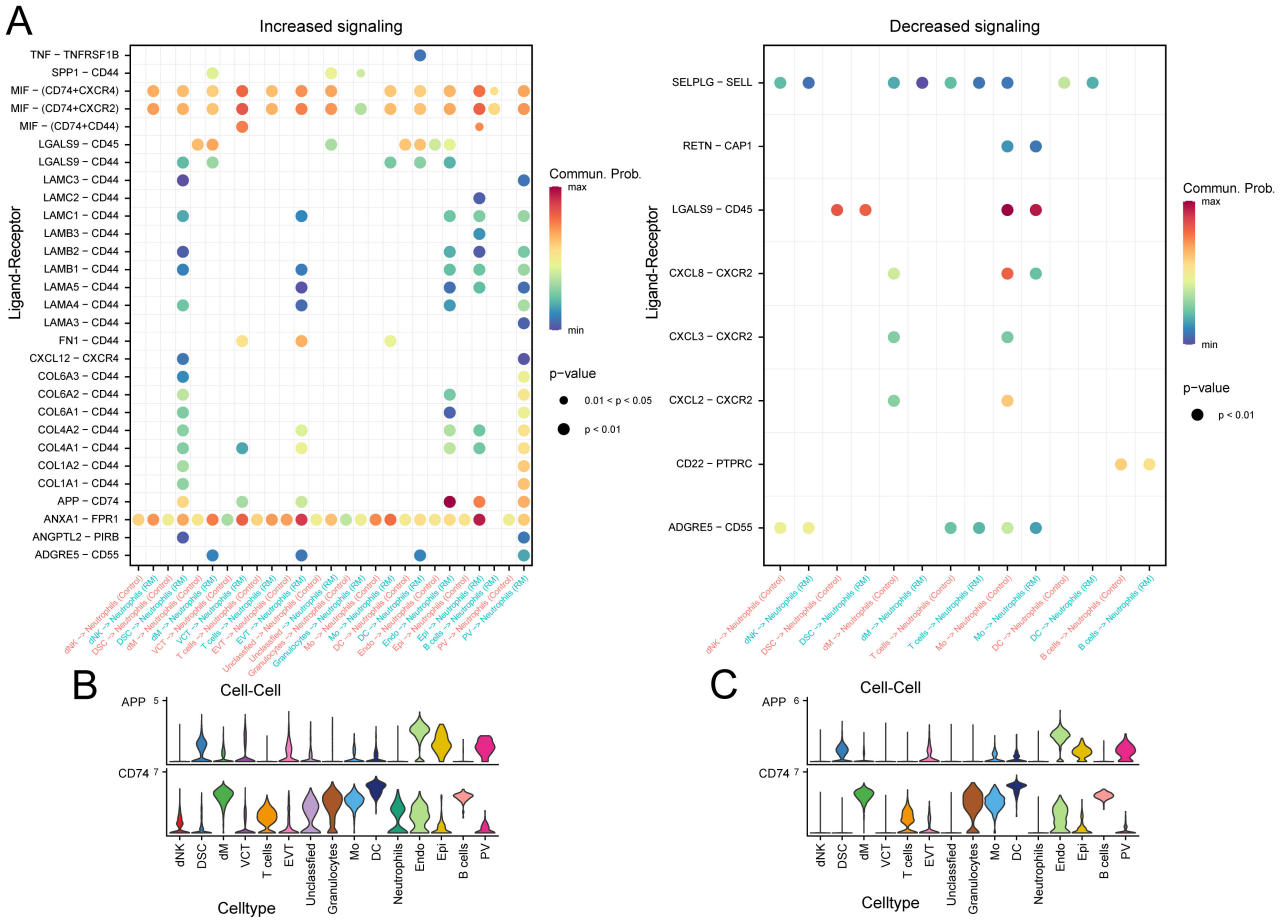
Specific pathway communication alterations involving APP–CD74. **(A)** Dot plot of ligand–receptor interactions between neutrophils and other cell types. **(B)** Expression of APP-derived ligand and CD74 receptor in RM samples. **(C)** Expression of APP-derived ligand and CD74 receptor in control samples.

Examining neutrophils as signal-receiving cells, we analyzed potential ligand-receptor pairs mediating communication between neutrophils and other cell types. In the RM group, substantial binding occurred between the APP ligand derived from Endo and its corresponding receptor, CD74, expressed on neutrophils. This interaction was absent in the control group (**Figure 5A**). Analysis of the expression patterns of the specific APP pathway ligand and receptors (CD74) showed that the receptor CD74 was highly expressed in neutrophils exclusively in the RM group (**Figures 5B, C**).

These results suggest that altered cell–cell communication, particularly the APP–CD74 axis, enhances neutrophil activation and may contribute to trophoblast dysfunction in RM.

### Pseudotime analysis

**Figure 6:**
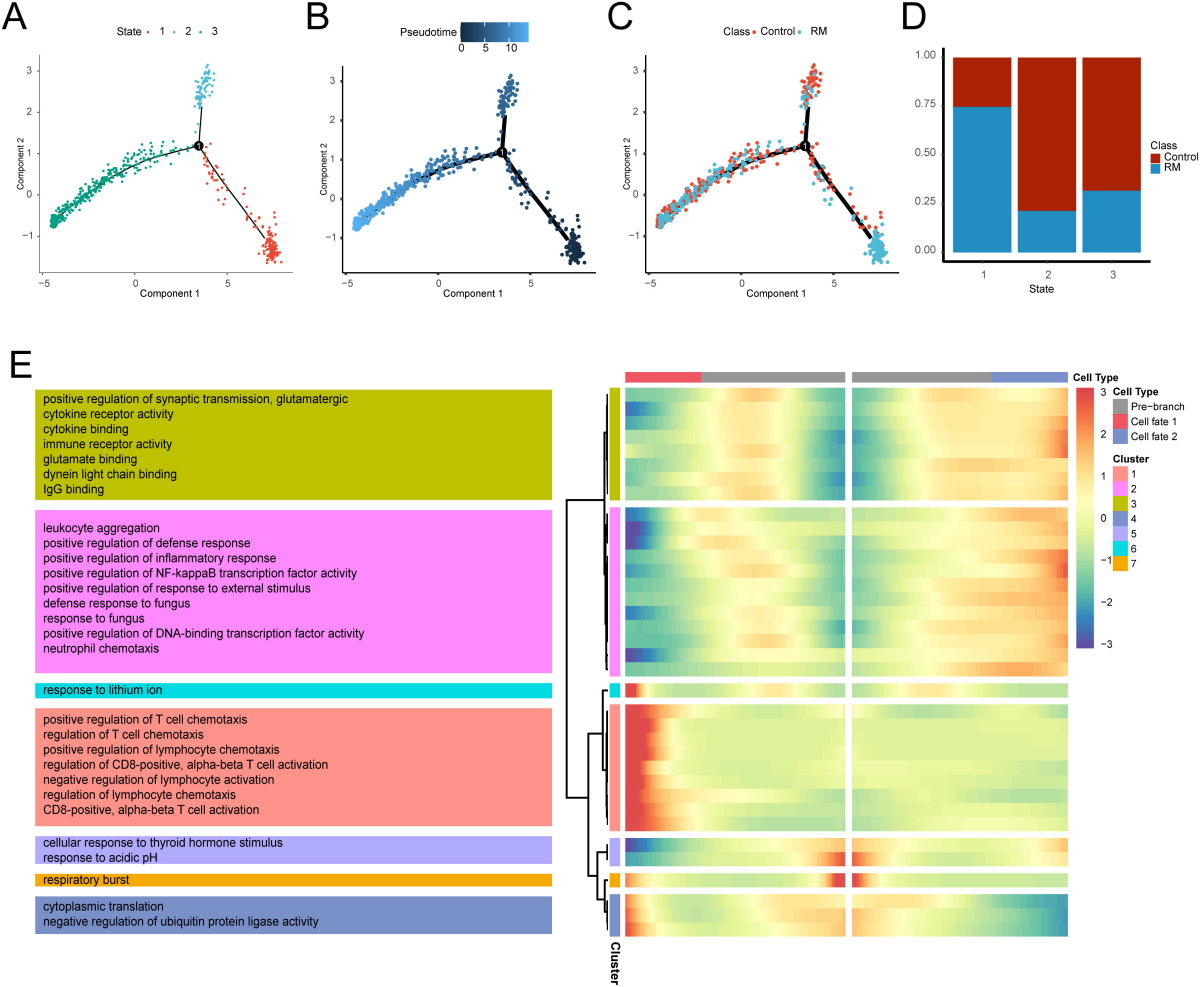
Transcriptional trajectory analysis of neutrophils states. **(A)** Neutrophil distribution across transcriptional states. **(B)** Neutrophil distribution along pseudotime. **(C)** Pseudotime-stratified distribution of RM vs. control neutrophils. **(D)** Cell proportion per transcriptional state. **(E)** Heatmap of branch-dependent DEGs and enriched GO pathways.

A pseudotemporal trajectory analysis was conducted for neutrophils to identify key gene expression programs governing neutrophil progression in RM. This analysis revealed three distinct transcriptional states: State 1 (trajectory initiation, predominantly RM-derived), State 3 (trajectory terminus), and State 2 (transitional state). The cellular composition across states is illustrated in (**Figures 6A–D**). A notable compositional transition occurred at Node 1. To understand the molecular mechanisms underlying this transition, branch-determinant genes were identified at Node 1. GO analysis indicated that pre-branch (State 1) highly-expressed genes were enriched in cellular response to thyroid hormone stimulus and respiratory burst pathways. Branch 2 (State 3) genes demonstrated enrichment for leukocyte aggregation, positive regulation of synaptic transmission, and glutamatergic pathways, while branch 1 (State 2) genes were associated with positive regulation of T cell chemotaxis, cytoplasmic translation, and response to lithium ion (**Figure 6E**), **Supplementary Table S9**). The analysis reveals a dynamic neutrophil trajectory in RM, marked by early immune activation and subsequent transitions toward pro-inflammatory states.

### KGs screening

Integrated analysis of transcriptomic datasets (GSE113790, GSE183555, GSE26787) identified 547 DEGs in RM versus controls (RM_vs_Control_DEGs, **Supplementary Table S10**; *p* < 0.05, |Log_2_FC| > 0.5). The differential gene distribution was visualized through a volcano plot, with a heatmap highlighting the top 5 up/down-regulated DEGs (**Supplementary Figures S3A, B**). Intersection analysis between transcriptomic DEGs and single-cell neutrophil DEGs (Ne_RM_vs_Control_scDEGs) revealed 7 KGs (**Supplementary Figure S3C**). KG correlations and group expression profiles are depicted in **Supplementary Figures S3D, E**. Functional enrichment analysis revealed significant pathway associations: GO terms included neutrophil-specific BP (neutrophil chemotaxis, neutrophil migration, granulocyte chemotaxis), CC (specific granule, tertiary granule), and MF (receptor ligand activity, signaling receptor activator activity, growth factor activity); KEGG pathway analysis showed enrichment in Hematopoietic cell lineage (**Supplementary Figures S3F, G**).The identification of neutrophil-associated key genes, including CXCL1 and CXCR1, highlights potential molecular participants within the neutrophil chemotactic signaling axis that may contribute to RM-associated immune dysregulation.

### KGs interaction network construction

PPI network analysis using GeneMANIA identified 20 genes functionally associated with the KGs (**Supplementary Figure S4A**; **Supplementary Table S11**). Functional enrichment analysis of the 27 co-expressed genes revealed significant GO enrichment in neutrophil-specific BP (neutrophil migration, granulocyte migration, and neutrophil chemotaxis), CC (specific granule, tertiary granule, and secretory granule membrane), and MF (CXCR chemokine receptor binding, receptor ligand activity, and chemokine activity), with KEGG pathway analysis showing enrichment in Legionellosis, Rheumatoid arthritis, and Amoebiasis (**Supplementary Figures S4B, C**). TRRUST database analysis identified 24 TFs interacting with 4 key genes, visualized via Cytoscape (**Supplementary Figure S4D**; **Supplementary Table S12**), while StarBase-derived RBP-mRNA regulatory network analysis identified 40 nodes (34 RBPs, 6 mRNAs) and 43 regulatory edges for 6 KGs (**Supplementary Figure S4E**). Network analyses further support the central role of these genes in regulating neutrophil function and immune dysregulation in RM.

### Diagnostic performance of KGs and model construction

**Figure 7:**
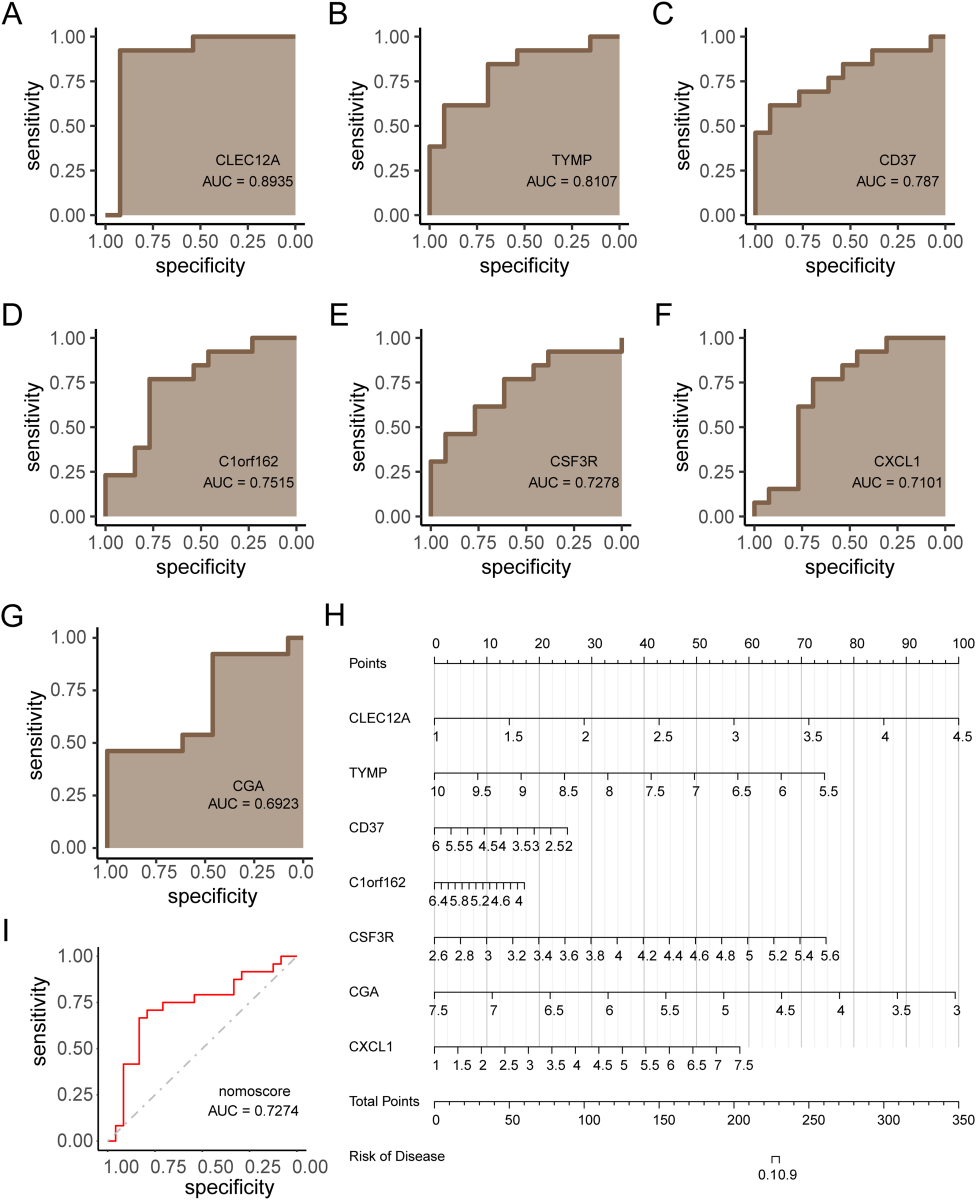
Diagnostic model construction and validation for RM. **(A–G)** ROC curves evaluating diagnostic performance of each key gene (KG). **(H)** Nomogram integrating KGs for RM prediction. **(I)** ROC validation of the nomogram in independent cohort GSE165004 (AUC > 0.7).

The diagnostic efficacy of the 7 KGs for RM was evaluated using ROC curves (**Figures 7A–G**), demonstrating strong discriminative capacity for each gene. A diagnostic nomogram incorporating these KGs was developed (**Figure 7H**). The predictive accuracy of this model was validated using the independent cohort GSE165004, with ROC analysis confirming robust diagnostic performance (**Figure 7I**, AUC > 0.7). The robust diagnostic performance of the KG-based nomogram suggests its potential clinical utility in identifying RM patients.

### Expression validation in AIDs

**Figure 8:**
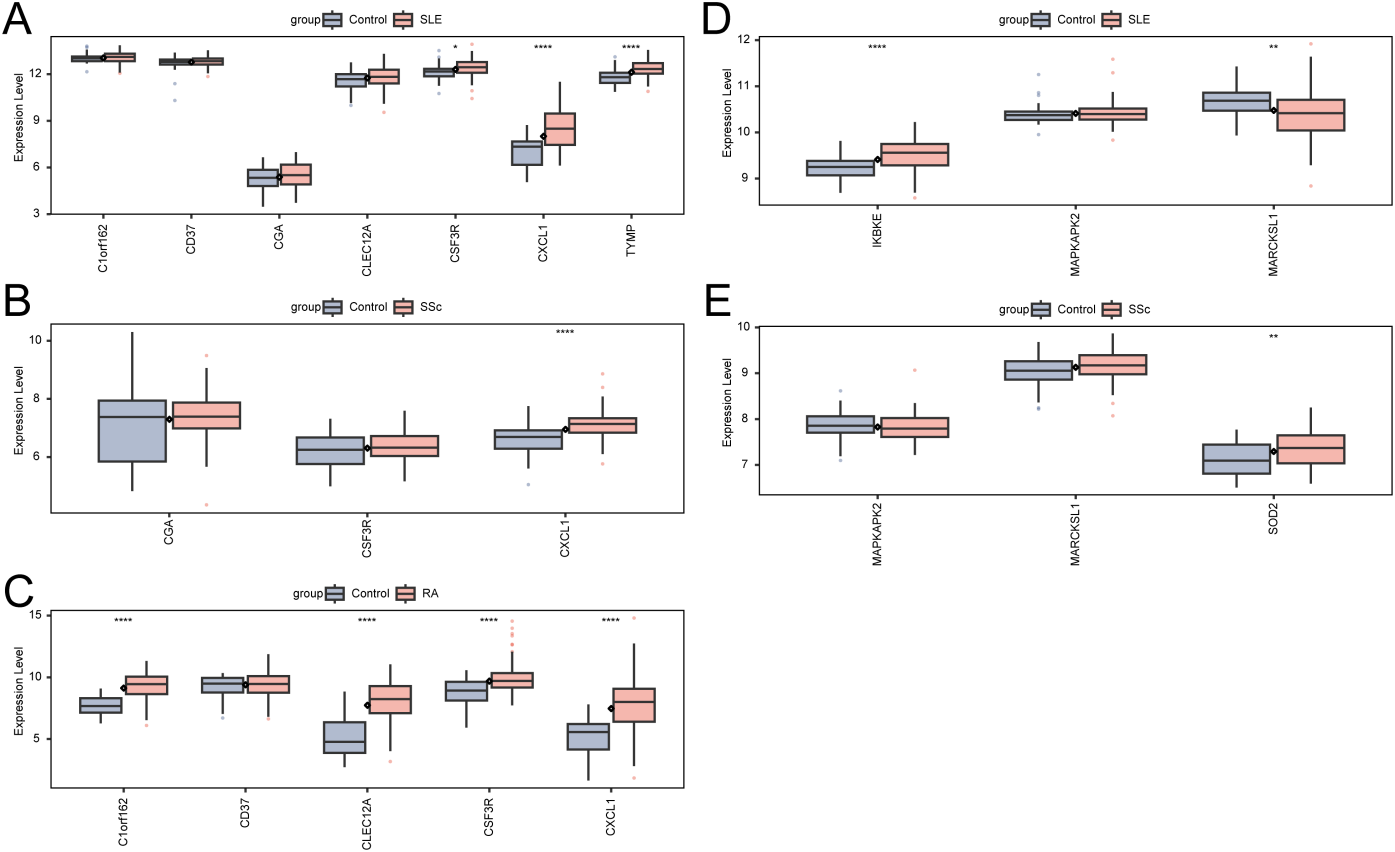
Expression of KGs and Neu-d marker genes in autoimmune diseases. **(A)** KG expression in SLE. **(B)** KG expression in SSc. **(C)** KG expression in RA. **(D)** Neu-d marker gene expression in SLE. **(E)** Neu-d marker gene expression in SSc.

To evaluate whether the neutrophil-associated KGs identified in RM were disease-specific or reflected broader autoimmune-driven inflammation, we conducted a stratified comparison across RA, SLE, and SSc cohorts (**Figures 8A–E**). After cohort-specific normalization and differential analysis, the expression profiles of RM-associated KGs were examined in each AID dataset independently. CXCL1 and selected KGs showed consistent upregulation across AIDs, suggesting shared immunoinflammatory features; however, several markers displayed stronger enrichment in RM than in AIDs, indicating potential RM-specific neutrophil signatures. These stratified comparisons helped delineate shared versus RM-preferential pathways and informed the downstream interpretation of disease relevance.

### Potential drug identification and molecular docking

**Figure 9:**
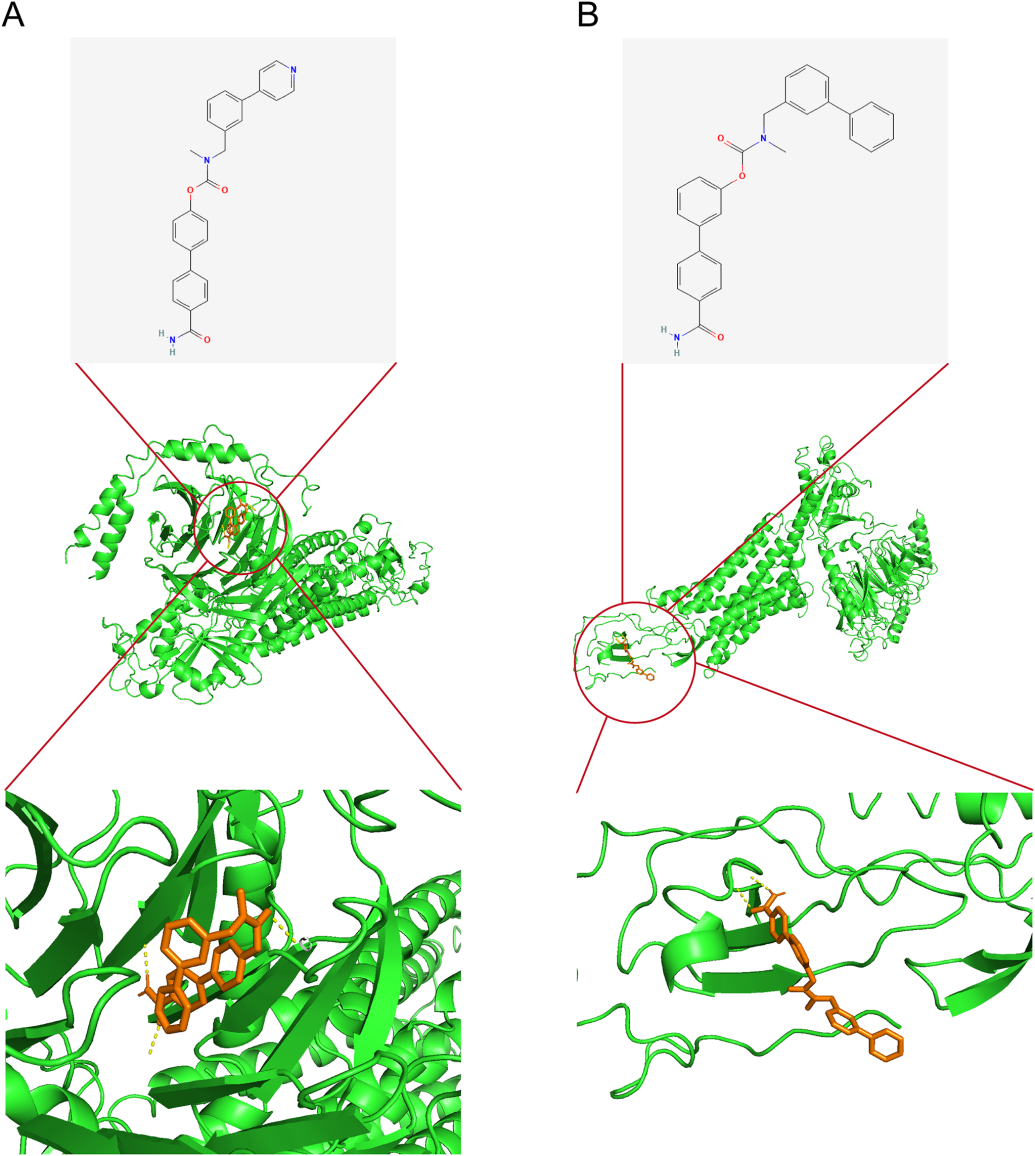
Candidate drug identification and molecular docking. **(A)** Docking configuration of CXCL1 with WWL70. **(B)** Docking configuration of CXCL1 with WWL123. Note: CXCL1–CXCR1 findings are exploratory and require functional confirmation in RM.

KGs were investigated as potential therapeutic targets using the DGIdb database to identify candidate drug molecules. Molecular docking simulations were performed (**Supplementary Table S13**), with WWL70 and WWL123 identified as high-affinity ligands for CXCL1 protein (interaction score >8). The binding energies (**Table 1**) confirmed strong protein-ligand interactions (Binding energy < -5 kcal/mol), indicating therapeutic potential for RM treatment. Molecular docking configurations were visualized in **Figures 9A, B**. These results identify candidate compounds targeting CXCL1, providing a rationale for therapeutic intervention in RM.

**Table 1:** Molecular docking affinity of identified candidate drug molecules and CXCL1.

Target	Drug	PDB ID	Binding energy(kcal/Mol)
CXCL1	WWL70	8k4o	-5.7
	WWL123		-5.5

### Immune-mediated trophoblast injury and ROS induction

**Figure 10:**
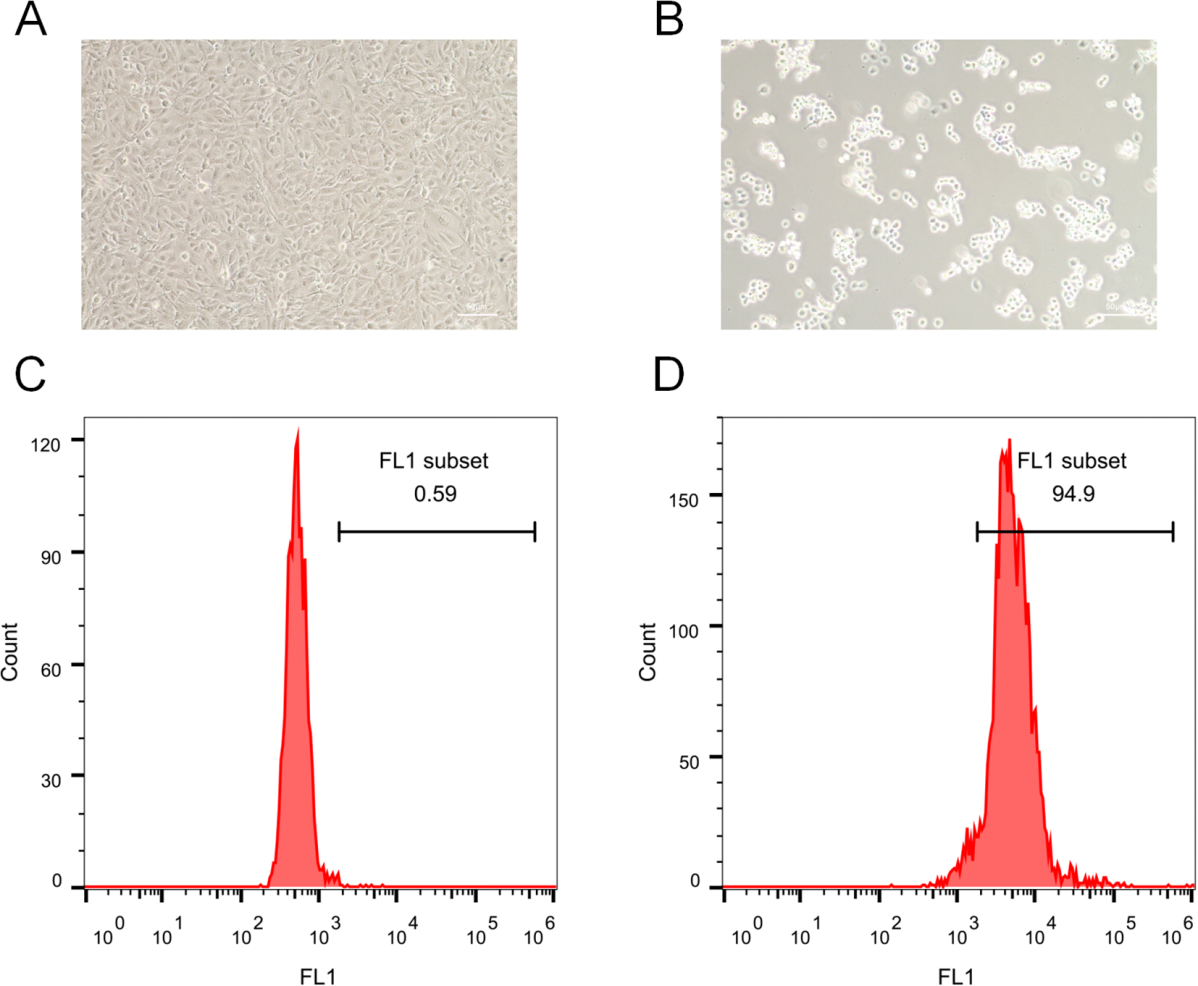
Immune-mediated trophoblast injury and ROS induction. **(A)** Morphology of control HTR-8/SVneo cells. **(B)** Morphology after anti-β_2_-GPI treatment. **(C)** Flow cytometry revealed 0.59% ROS-positive cells in controls. **(D)** Anti-β_2_-GPI treatment greatly increased ROS abundance, with 94.9% ROS-positive cells.

To provide experimental support for our computational findings implicating neutrophil-associated inflammatory and ROS-related pathways in RM, we performed an *in-vitro* trophoblast injury assay using HTR-8/SVneo cells stimulated with anti-β_2_-GPI antibody to mimic RM-associated immune activation. Light-microscopy examination showed that control cells exhibited normal morphology and high density (**Figure 10A**), whereas anti-β_2_-GPI–treated cells displayed marked shrinkage, detachment, and reduced density (**Figure 10B**), consistent with trophoblast injury. Intracellular ROS levels were quantified by flow cytometry: only 0.59% of control cells were ROS-positive (**Figure 10C**), whereas anti-β_2_-GPI stimulation increased ROS-positive cells to 94.9% (**Figure 10D**). These findings indicate that immune stimulation triggers pronounced oxidative stress and impairs trophoblast viability.

Importantly, these observations are aligned with our single-cell transcriptomic and IREA analyses, which demonstrated TNF-α–driven neutrophil polarization and elevated oxidative-stress signatures in RM. Although this assay models the downstream impact of immune activation rather than direct neutrophil–trophoblast contact, it supports the predicted role of inflammatory microenvironment–induced oxidative stress as a contributor to trophoblast dysfunction in RM. Future studies employing direct co-culture or transwell models of neutrophils and trophoblasts will be required to further define cellular interactions.

## Discussion

This study characterizes the decidual immune microenvironment in RM using bulk and single-cell transcriptomics, supported by experimental validation. We identified a consistent expansion of neutrophils in RM, accompanied by a TNF-α–driven polarized phenotype enriched for oxidative stress and antigen presentation pathways. Intercellular communication analysis revealed enhanced crosstalk, particularly via the APP–CD74 axis targeting neutrophils, while pseudotime trajectories indicated early immune activation and progressive pro-inflammatory transitions. Integrative multi-omics further highlighted seven neutrophil-associated KGs, especially CXCL1, which exhibited robust diagnostic performance and consistent dysregulation across AIDs. Network analyses confirmed their immunoregulatory centrality, and molecular docking suggested candidate drugs targeting CXCL1. Finally, *in vitro* experiments demonstrated that immune-mediated oxidative stress impaired trophoblast viability, corroborating computational predictions. Collectively, these findings establish neutrophil-centered immune dysregulation as a pivotal mechanism in RM pathogenesis.

Neutrophils, traditionally seen as short-lived innate immune cells, now play roles in chronic inflammation, autoimmunity, and tissue remodeling. In RM, they not only increase in abundance but also acquire a reprogrammed phenotype. By integrating cell–cell communication and trajectory analyses, we provide mechanistic insights into how these neutrophils may disrupt trophoblast function and compromise pregnancy maintenance. Importantly, the consistency of CXCL1 dysregulation across RM and AIDs suggests shared immune pathways, raising the possibility that RM represents a manifestation of systemic immune dysregulation.

TNF-α, a pro-inflammatory cytokine, plays a central role in RM by activating immune cells, including neutrophils, which mediate inflammation. Our study confirms and extends these findings by showing that TNF-α induces a polarized neutrophil phenotype characterized by increased oxidative stress and antigen presentation. This aligns with findings from other studies showing that excessive neutrophil activation leads to tissue damage through ROS production and matrix remodeling, processes known to impair placental development and function (5–26).

Additionally, our cell–cell communication analysis highlighted the APP–CD74 axis as a prominent ligand–receptor interaction involving neutrophils in RM. Although direct evidence linking APP–CD74 signaling to neutrophil activation in RM is currently lacking, CD74 has been implicated in pregnancy-related disorders. For example, reduced CD74 expression in placental macrophages has been associated with impaired macrophage–trophoblast interactions and heightened inflammatory responses in preeclampsia (27). In addition, APP–CD74 communication has been reported to mediate immune cell crosstalk in models of tissue injury and fibrosis (28). These findings suggest that APP–CD74 signaling contributes to neutrophil activation and trophoblast dysfunction in RM, warranting further validation. Although CD74 has been reported to mediate immunosuppressive signaling in certain neutrophil subsets, particularly under steady-state or tumor-associated conditions, our findings indicate that APP-CD74 engagement may exert a pro-inflammatory function within the TNF-α-enriched decidual microenvironment of RM. Supporting evidence from macrophage and endothelial models shows that APP-CD74 interaction activates NF-κB and MAPK pathways (27, 28). Given the elevated TNF-α and oxidative stress observed in RM, it is plausible that CD74 signaling acts as a co-stimulatory receptor, amplifying neutrophil activation and promoting trophoblast injury rather than suppression. This context-dependent duality of CD74 may reflect its functional plasticity across immune environments.

Pseudotime analysis showed early immune activation in RM, with neutrophils prematurely activated, contributing to the chronic inflammation seen in pregnancy loss. Similar findings have been observed in AIDs, where neutrophils are often in an activated state, further supporting the idea that RM may involve systemic immune dysregulation (29).

These insights have clear translational implications. The identification of CXCL1 and related KGs as diagnostic markers highlights opportunities for developing predictive models to identify women at high risk of RM. Furthermore, molecular docking predictions suggest therapeutic avenues for pharmacological modulation of neutrophil activity, although preclinical and clinical validation will be essential. CXCL1 has been identified as a key chemokine in several inflammatory disorders, including AIDs, and targeting this pathway could represent a novel therapeutic strategy for RM management (30).

Although our integrative analyses identified CXCR1 as part of the neutrophil-related gene network in RM, its role should be interpreted with caution. The current data suggest an association rather than causation, as no direct experimental evidence yet links CXCR1-mediated signaling to trophoblast injury. Previous reports in autoimmune and inflammatory contexts have demonstrated CXCR1 involvement in neutrophil migration and activation, supporting its potential relevance. Nonetheless, functional studies are needed to determine whether CXCR1 contributes mechanistically to RM pathogenesis. Therefore, we refer to CXCR1 as a candidate molecule within the CXCL1–CXCR1 chemotactic axis rather than as a confirmed therapeutic target.

Importantly, our *in-vitro* validation provides preliminary biological support for the computational observation that RM is associated with heightened inflammatory and oxidative stress responses. Stimulation of HTR-8/SVneo trophoblasts with anti-β_2_-GPI antibody—used here to mimic RM-related immune activation—induced marked morphological injury and a dramatic increase in intracellular ROS levels, consistent with impaired trophoblast viability. These findings align with the neutrophil-associated TNF-α–polarized signatures and ROS-enrichment identified by scRNA-seq and IREA. Because this model captures downstream immune-activation effects rather than direct neutrophil–trophoblast interaction, the data should be interpreted as indirect validation of the inflammatory oxidative milieu predicted computationally. Nonetheless, the phenotypic injury observed in trophoblasts reinforces the biologic plausibility that immune dysregulation contributes to placental dysfunction in RM.

Several limitations warrant consideration in this study. Although we employed high-resolution single-cell sequencing, the sample sizes of the cohorts used for analysis remain relatively small. While these datasets are comprehensive, they may not fully capture the heterogeneity of RM across different populations, and further studies with larger, more diverse samples are needed to validate our findings. In addition, while our computational analysis suggests several promising drug targets, these predictions require experimental validation. Our molecular docking results, while promising, have yet to be confirmed *in vitro* and *in vivo*.

Although *in-vitro* activation of trophoblasts corroborates computational predictions of ROS-associated tissue injury, the assay does not provide direct evidence of neutrophil-derived factors acting on trophoblasts. Moreover, our anti-β_2_-GPI–based model was intended to mimic the immune-activation environment observed in RM rather than to directly assess neutrophil–trophoblast interactions. Thus, additional studies are needed to explore the direct contribution of neutrophils *in vivo*. Future experiments involving coculture or transwell systems with isolated neutrophils and trophoblasts will be essential to determine whether activated neutrophils directly impair trophoblast function, and to clarify signaling mechanisms such as the APP–CD74 axis in RM. Finally, the mechanisms underlying neutrophil-mediated trophoblast dysfunction remain only partially understood, and further mechanistic studies are essential to clarify the specific role of neutrophils in pregnancy maintenance and loss.

Looking forward, longitudinal profiling of immune dynamics throughout gestation will be important to clarify the temporal evolution of neutrophil states in RM. Functional experiments in animal models and larger patient cohorts will be critical to establish causality and to evaluate the therapeutic efficacy of targeting neutrophil polarization or APP–CD74 signaling. Translational studies should also assess the predictive utility of CXCL1 and other KGs in clinical practice, with the goal of developing personalized diagnostic tools. Ultimately, integrating single-cell immunology with precision-medicine approaches may enable the development of novel therapeutic strategies to prevent pregnancy loss and improve maternal health.

## Conclusion

In conclusion, our study delineates a neutrophil-driven immune mechanism underlying RM, integrating computational predictions with biological validation. The discovery of a TNF-α–polarized neutrophil phenotype, disrupted intercellular communication, and key diagnostic markers such as CXCL1 provides novel insights into RM pathogenesis and highlights opportunities for early diagnosis and therapeutic intervention. Looking ahead, longitudinal and translational studies will be essential to validate these findings and to advance precision medicine approaches for RM.

## Data Availability

The original contributions presented in the study are included in the article/**Supplementary Material**. Further inquiries can be directed to the corresponding authors.
