# Supplementary figures and images for: Neutrophil-mediated immune dysregulation in recurrent miscarriage: implications of APP–CD74 signaling and CXCL1 as diagnostic biomarkers
Source: Front Immunol. 2025 Dec 4;16:1707497. doi: 10.3389/fimmu.2025.1707497 (PMC12711488; doi:10.3389/fimmu.2025.1707497)

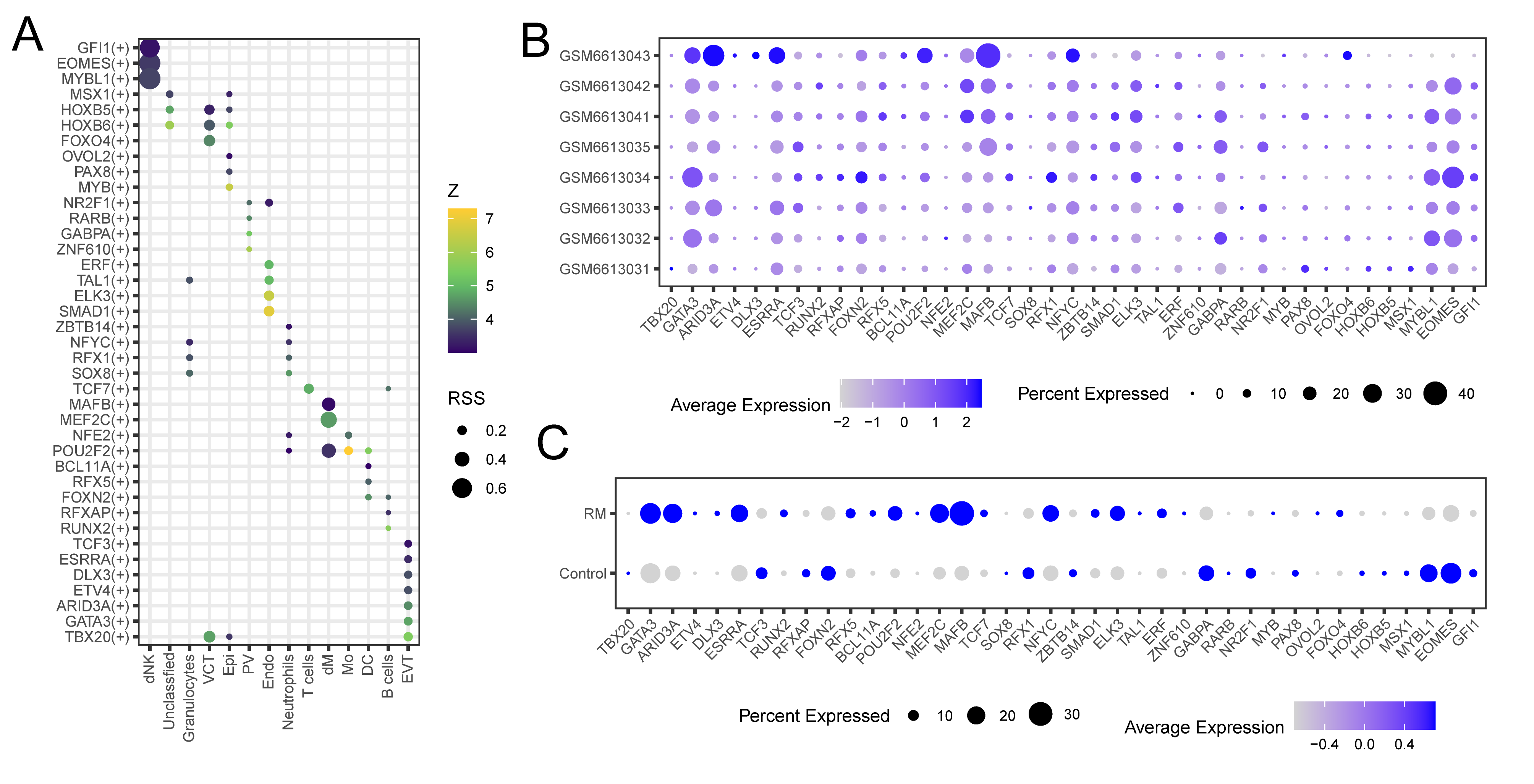

Supplement: Supplementary file 2 [file Image1.tif]

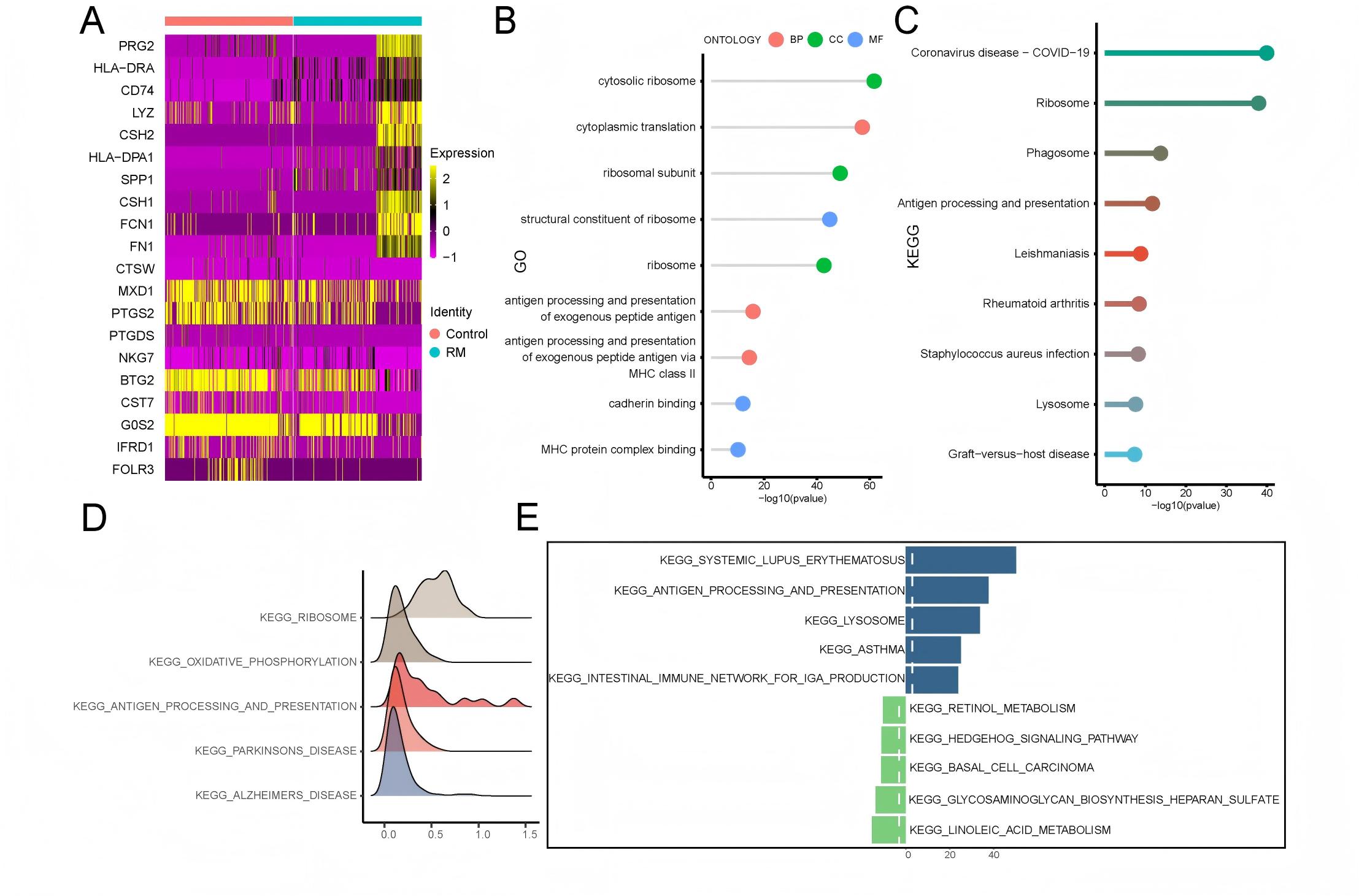

Supplement: Supplementary file 3 [file Image2.jpeg]

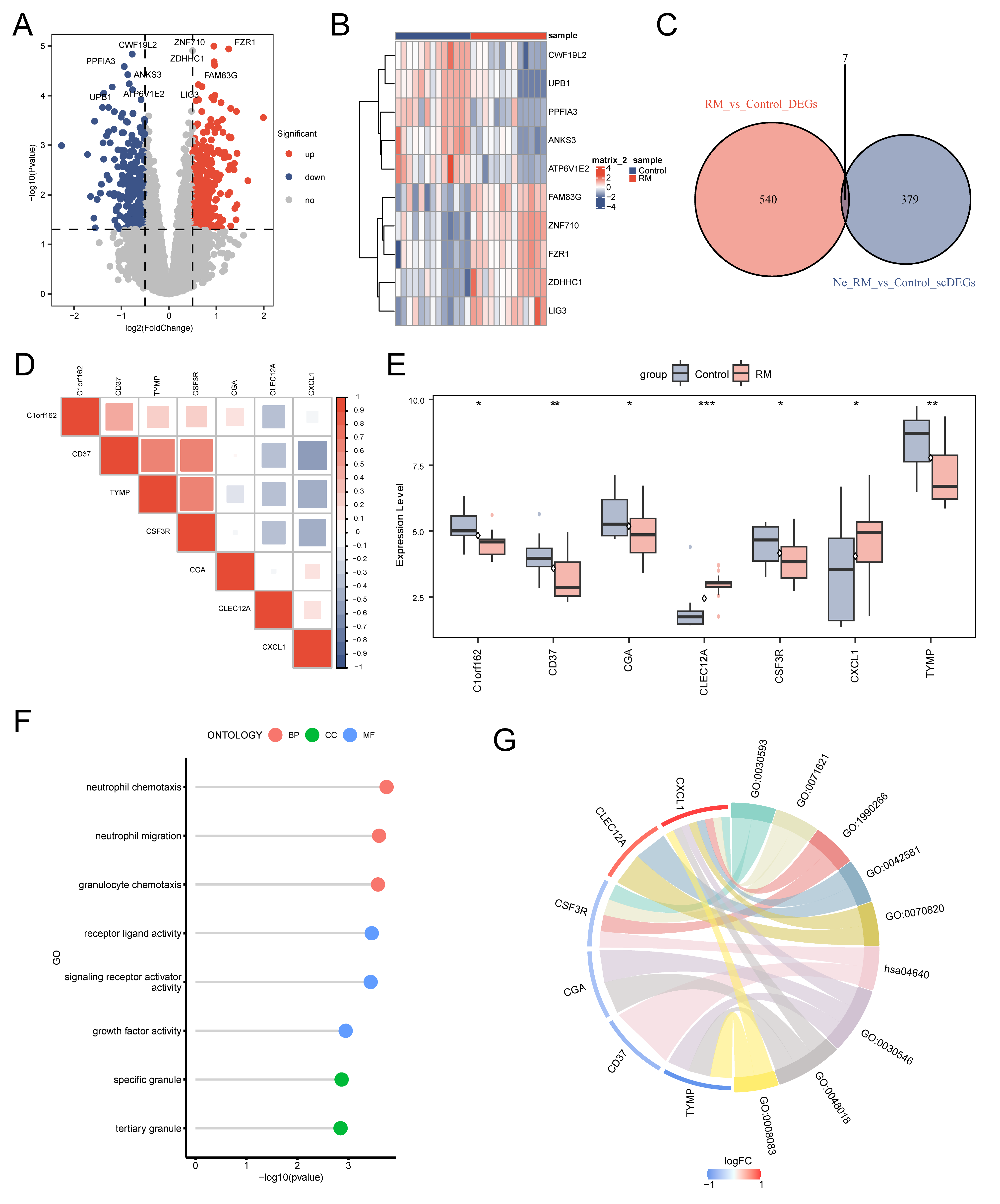

Supplement: Supplementary file 4 [file Image3.tif]

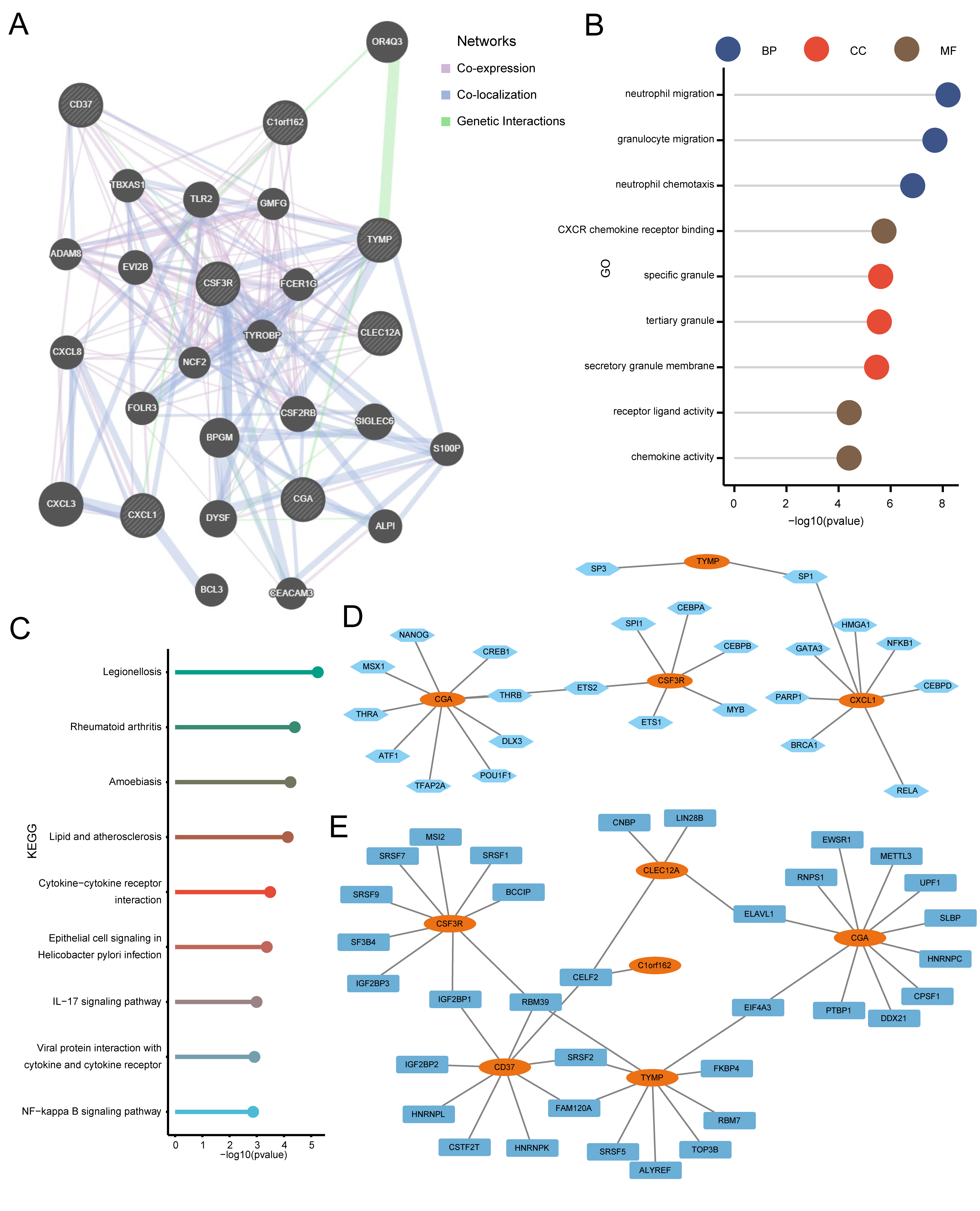

Supplement: Supplementary file 5 [file Image4.tif]
